# Coffee and Tea Consumption Impact on Amyotrophic Lateral Sclerosis Progression: A Multicenter Cross-Sectional Study

**DOI:** 10.3389/fneur.2021.637939

**Published:** 2021-07-28

**Authors:** Aliona Cucovici, Andrei Ivashynka, Andrea Fontana, Sergio Russo, Letizia Mazzini, Jessica Mandrioli, Vitalie Lisnic, Dafin Fior Muresanu, Maurizio Angelo Leone

**Affiliations:** ^1^Department of Medical and Surgical Sciences, University of Foggia, Foggia, Italy; ^2^Fondazione Istituto di Ricovero e Cura a Carattere Scientifico (IRCCS) Casa Sollievo della Sofferenza, San Giovanni Rotondo, Italy; ^3^Department of Neuromuscular Diseases, “Diomid Gherman” Institute of Neurology and Neurosurgery, Chisinau, Moldova; ^4^Department of Health Sciences, University of Eastern Piedmont, Novara, Italy; ^5^Department of Rehabilitation and Functional Recovery, Istituti Clinici Scientifici (ICS) Maugeri, Istituto di Ricovero e Cura a Carattere Scientifico (IRCCS) Bari, Bari, Italy; ^6^Innovation and Research Unit, Fondazione IRCCS Casa Sollievo della Sofferenza, San Giovanni Rotondo, Italy; ^7^Department of Neurology, Amyotrophic Lateral Sclerosis (ALS) Center, Maggiore della Carità Hospital, Università del Piemonte Orientale, Novara, Italy; ^8^Department of Neurosciences, University Hospital of Modena, Modena, Italy; ^9^Department of Neurology nr.1, “Nicolae Testemitanu” State University of Medicine and Pharmacy, Chisinau, Moldova; ^10^Department of Clinical Neurosciences, “Iuliu Hatieganu” University of Medicine and Pharmacy, Cluj-Napoca, Romania

**Keywords:** amyotrophic lateral sclerosis, coffee, tea, risk factors, protective factors, rate of disease progression

## Abstract

**Background/objectives:** Amyotrophic lateral sclerosis (ALS) is a devastating and still untreatable motor neuron disease. The causes of ALS are unknown, but nutritional factors may impact the rate of disease progression. We aimed to ascertain the influence of coffee and tea consumption on ALS progression rate.

**Subjects/methods:** In this multicenter cross-sectional study, we recruited 241 patients, 96 females, and 145 males; the mean age at onset was 59.9 ± 11.8 years. According to El Escorial criteria, 74 were definite ALS, 77 probable, 55 possible, and 35 suspected; 187 patients had spinal onset and 54 bulbar. Patients were categorized into three groups, according to their ΔFS (derived from ALS Functional Rating Scale-Revised score and disease duration from onset): slow (81), intermediate (80), and fast progressors (80).

**Results:** Current coffee consumers were 179 (74.3%), 34 (14.1%) were non-consumers, and 22 (9.1%) were former consumers, whereas six (2.5%) consumed decaffeinated coffee only. The log-ΔFS was weakly correlated with the duration of coffee consumption (*p* = 0.034), but not with the number of cup-years, or the intensity of coffee consumption (cups/day). Current tea consumers were 101 (41.9%), 6 (2.5%) were former consumers, and 134 (55.6%) were non-consumers. Among current and former consumers, 27 (25.2%) consumed only green tea, 51 (47.7%) only black tea, and 29 (27.1%) both. The log-ΔFS was weakly correlated only with the consumption duration of black tea (*p* = 0.028) but not with the number of cup-years.

**Conclusions:** Our study does not support the hypothesis that coffee or tea consumption is associated with the ALS progression rate.

## Introduction

Amyotrophic lateral sclerosis (ALS) is an untreatable neurodegenerative disease characterized by progressive degeneration of motor neurons in the spinal cord and motor cortex. The main clinical predictors of progression are age and site of onset, diagnostic delay, and the Amyotrophic Lateral Sclerosis Functional Rating Scale-Revised (ALSFRS-R) baseline score (1). The role of lifestyle factors, such as physical activity, smoking, diet, alcohol intake, coffee, and tea consumption, on ALS progression is unclear (2) Similar to other neurodegenerative diseases (3, 4), some potentially modifiable lifestyle factors could impact ALS progression, suggesting possible clues to understand its pathogenesis and possible interventions. Coffee and tea are the most consumed methylxanthine-containing beverages worldwide, and their effects on the nervous system have been widely explored (5, 6). Caffeine is a major active principle in coffee and tea, antagonizing the adenosine A2A receptors in the brain and defending the motor neurons against excitotoxicity (7). Coffee and tea consumptions were studied for their possible impact on the risk of ALS onset, although most studies are negative (8, 9).

Still, there are no studies regarding the influence of coffee and tea consumption on ALS progression. The risk factors for progression may not be the same as for disease susceptibility; we aimed to assess a possible role of lifetime coffee and tea consumption on ALS progression.

## Materials and Methods

The study was designed as a cross-sectional multicenter study. It was conducted in three Centers in Italy—San Giovanni Rotondo (Coordinating Center), Novara, and Modena—one in the Republic of Moldova (Chisinau), and one in Romania (Cluj-Napoca). The study was approved by the Institutional Review Boards of the coordinating Center (N96/CE/2016) and the other four Centers. Written informed consent was obtained from all participants.

### Patients

Patients were recruited from October 2016 to January 2020, in different periods in each Center. Inclusion criteria were age higher than 18 years, diagnosis according to the El Escorial criteria (10), and consecutive inpatients and outpatients with a new or already made clinical diagnosis of ALS. Patients with tracheostomy or receiving mechanical ventilation, with percutaneous endoscopic gastrostomy, or who did not sign the informed consent form were excluded from the study.

### Data Collection and Disease Progression Assessment

For each patient, we collected demographics (date of birth, gender, and education) and clinical data [date of onset and diagnosis, site of onset, diagnostic category according to El Escorial criteria, BMI, forced vital capacity (FVC%), and treatment]. Disease severity was estimated through ALSFRS-R, which evaluates the severity of the disease through a 12-item questionnaire (11). The rate of disease progression (ΔFS) at recruitment was calculated by dividing the ALSFRS-R total score by symptom duration applying the formula: ΔFS = (48-total ALSFRS-R at visit)/symptom duration in months (12). The date of disease onset was determined on subjective complaints and information confirmed from relatives and clinical charts.

### Exposure Assessment

Cigarette smoking and alcohol consumption histories were evaluated with the “Questionnaire of Lifestyle,” which is part of the European Prospective Investigation into Cancer and Nutrition project study (13, 14). We defined three categories of smoking status at recruitment in relation to disease onset: non-smokers were those who had smoked <100 cigarettes up to the time of the interview (15) or stopped smoking at least 6 months before the disease onset; current smokers were those who had smoked >100 cigarettes and were still smoking at the time of the interview or within 6 months of the interview; and former smokers were those who had smoked >100 cigarettes and had stopped smoking after disease onset, but at least 6 months before the time of the interview. Similarly, alcohol drinking status was defined as follows: non-drinkers were those who had drunk less than one standard alcohol drink/month or had stopped drinking at least 6 months before the disease onset; current drinkers were those who had drunk more than one standard alcohol drink/month and were still drinking at recruitment; and former drinkers were those who had drunk more than one standard alcohol drink/month and had stopped drinking after disease onset, but at least 6 months before the interview (2).

Coffee and tea consumption histories were evaluated with a questionnaire built in analogy to the “Questionnaire of Lifestyle” asking patients whether they consumed or had consumed in the past coffee (regular or decaffeinated) or tea (green or black tea) and, if so, how many cups per day. One standard unit is equivalent to one cup of coffee or tea (about 30 and 170 ml, respectively) (16). Detailed information was obtained regarding coffee and tea consumption at the ages of 20, 30, 40, 50, 60, 70, and over up to the participants' current age. For each beverage, we obtained age at onset of consumption and cessation (for former consumers). For coffee and tea consumption, we defined three categories of consuming status at recruitment in relation to disease onset: non-consumer were those who had never consumed more than one unit/month or stopped consuming at least 6 months before disease onset; current consumer were those who consumed beverages at least monthly for 6 months or longer and were still consuming at recruitment, or within 6 months of the interview; and former consumer were those who had consumed beverages at least monthly for 6 months or longer and had stopped consuming after disease onset, but at least 6 months prior of recruitment.

For each current or former consumer, a cumulative lifetime load was computed for each beverage as the weighted sum of the number of cups consumed per year within each decade (six age periods), with weights equal to the number of years spent consuming in the decade (cup-years). This is the measure of the amount that a person has consumed over a lifetime and was computed by dividing the cumulative lifetime load by 365.25. We also calculated two measures of average daily consumption (cups/day): the lifetime intensity (17) during the lifetime was calculated as the lifetime load divided by the number of years spent drinking during a lifetime (i.e., coffee or tea consumption duration in years), and the intensity at the interview, calculated as the number of cups/day drunk at the time of the interview.

### Questionnaire

The questionnaire was designed in Italian, then translated in Romanian by a mother-language translator, and back-translated by an Italian mother-language translator. Two raters, previously trained in the use of the questionnaire and blinded to the patients' clinical status interviewed patients in a dedicated room. To evaluate the reliability of the questionnaire, two pairs of raters interviewed healthy subjects or patients with neurological diseases before the study started (40 in Chisinau and 25 in San Giovanni Rotondo). The sequence of interviews was randomized, and the randomization list was concealed. Each rater did the interviews on at least 1 day and no more than 7 days apart; this was considered a sufficient time window for the subjects being unable to remember their answers and not to change their consumption habits. Agreement between two raters for consumption (yes/no) was calculated with Cohen's kappa statistics (18) and was 0.95/1.0 for coffee/tea in Chisinau and 0.90/0.95 in San Giovanni Rotondo. Agreement for continuous variables was determined with the intraclass correlation coefficient (19) and was 0.99/1.0 for coffee/tea duration and 0.65/0.94 for coffee/tea cup-year.

### Statistical Analysis

The patients' characteristics are reported as mean ± standard deviation, or median with interquartile range (IQR), depending on their distribution, for continuous variables, and with absolute and relative frequencies (%) for categorical variables. The normality of continuous variables distribution was checked by the Q–Q plot and the Shapiro–Wilk test. In the presence of right-skewed continuous variables, statistical analyses were performed on log values. Comparisons between two categorical variables were assessed by chi-square or Fisher exact tests, whereas comparisons between a continuous and a categorical variable were assessed by univariable and multivariable ANOVA models. Pairwise comparisons between groups of the categorical variables were performed, and, if necessary, least-square means of the dependent variable (along with their 95% confidence interval) were estimated for each level of the categorical variable. The standardized mean difference was further reported to describe clinical characteristics and was computed as the average of all possible standardized mean differences across pairwise comparisons. Correlation between two continuous variables was assessed by the Pearson correlation coefficient.

To visually assess the relationship between drink dose (i.e., cup-year) and ΔFS or duration of drink consumption, boxplots and scatterplots with fitted regression line were depicted into a plot matrix. To detect all clinical, demographical, pathological, treatment, and lifestyle variables, which were mostly associated with (log-transformed) ΔFS, the conditional random forest (RF) algorithm (20) with 100,000 trees was performed. The RF is a popular machine learning tool that assesses the relationship between a dependent variable and a set of covariates in a (non-parametric) tree-based fashion. An important feature of RF is that it provides a rapidly computable internal measure of variable importance (VIMP) that can be used to rank variables. The VIMP produced by a conditional RF was not affected by the correlation structure of all the included covariates. Formally, a VIMP of a specific covariate is defined as the sum of the decrease in prediction error values when a tree of the forest splits by that covariate. The more a tree relies on a variable to make predictions, the more important it is for that tree. The relative importance is the VIMP divided by the highest VIMP value. A two-sided *p*-value < 0.05 was considered for statistical significance. All statistical analyses were performed using SAS Release 9.4 (SAS Institute, Cary, NC, USA). Conditional random forests and plots were performed using R Foundation for Statistical Computing (version 3.6, packages: party, ggplot2).

## Results

We recruited 241 patients, 145 men and 96 women, with a sex ratio of 1.5:1. Onset was in the spinal district in 187 (77.6%) and bulbar in 54 (22.4%). The mean age was 59.9 ± 11.8 years at onset and 62.4 ± 11.1 at recruitment. The median time elapsed between disease onset to recruitment was 20 months (range 1.7–273). According to El Escorial criteria, 74 (30.7%) patients were categorized as definite ALS, 77 (32.0%) as probable, 55 (22.8%) as possible, and 35 (14.5%) as suspected. Other demographic and clinical characteristics are shown in Table 1. The ALSFRS-R score ranged from 10 to 48, with a mean of 34.9 ± 8.3. The ΔFS score ranged from 0 to 5.3, with a median of 0.56 (IQR: 0.25–1.05). Patients were categorized into tertiles according to the ΔFS distribution: (a) ≤ 0.333 (slow progressors), (b) 0.334–0.875 (intermediate progressors); and (c) >0.875 (fast progressors). Table 1 shows clinical characteristics according to ΔFS tertiles. Slow progressors were younger at disease onset and recruitment, had less frequently bulbar onset and a diagnosis of definite ALS, had a longer diagnostic delay, and had a better FVC% (Table 1).

**Table 1:** Clinical variables overall and according to the tertiles of ΔFS distribution.

**Variable**	**Category**	**All (*N* = 241)**	**I: Slow progression rate of disease (*N* = 81)**	**II: Intermediate progression rate of disease (*N* = 80)**	**III: Fast progression rate of disease (*N* = 80)**	***p*-value**	**SMD**
Country—*N* (%)	Italy	206 (85.5)	71 (87.7)	67 (83.8)	68 (85.0)	0.773	0.074
	Moldova and Romania	35 (14.5)	10 (12.3)	13 (16.2)	12 (15.0)		
Gender—*N* (%)	Males	145 (60.2)	53 (65.4)	44 (55.0)	48 (60.0)	0.401	0.143
	Females	96 (39.8)	28 (34.6)	36 (45.0)	32 (40.0)		
Age at interview (years)	Mean ± SD	62.4 ± 11.0	59.8 ± 12.3	63.6 ± 10.4	63.9 ± 9.8	0.032	0.241
Age at onset (years)	Mean ± SD	59.9 ± 11.8	54.6 ± 12.9	62.0 ± 10.5	63.2 ± 9.8	<0.001	0.502
Diagnostic delay (years)	Median (range)	0.9 (0.1–15.8)	1.7 (0.1–15.8)	0.8 (0.1–4.1)	0.5 (0.1–1.8)	<0.001^*^	1.020^*^
Education (years)	Mean ± SD	10.4 ± 4.4	11.1 ± 4.4	10.6 ± 4.3	9.5 ± 4.2	0.058	0.248
Site of onset—*N* (%)	Spinal	187 (77.6)	71 (87.7)	53 (66.2)	63 (78.8)	0.005	0.349
	Bulbar	54 (22.4)	10 (12.3)	27 (33.8)	17 (21.2)		
El Escorial ALS—*N* (%)	Definite	74 (30.7)	16 (19.8)	25 (31.2)	33 (41.2)	0.014	0.460
	Possible	55 (22.8)	23 (28.4)	23 (28.7)	9 (11.2)		
	Probable	77 (32.0)	26 (32.1)	23 (28.7)	28 (35.0)		
	Suspected	35 (14.5)	16 (19.8)	9 (11.2)	10 (12.5)		
FVC—*N* (%)	<80%	88 (43.8)	20 (29.0)	32 (47.1)	36 (56.2)	0.005	0.379
	≥80%	113 (56.2)	49 (71.0)	36 (52.9)	28 (43.8)		
BMI at interview—*N* (%)	<18.5	15 (6.2)	5 (6.2)	4 (5.0)	6 (7.5)	0.967	0.083
	18.5–24.9	121 (50.2)	42 (51.9)	40 (50.0)	39 (48.8)		
	≥25	105 (43.6)	34 (42.0)	36 (45.0)	35 (43.8)		
ALSFRS-R	Mean ± SD	34.9 ± 8.3	38.8 ± 6.9	35.2 ± 7.5	30.6 ± 8.4	<0.001	0.713
Riluzole—*N* (%)	Yes	129 (53.5)	41 (50.6)	47 (58.8)	41 (51.2)	0.517	0.109
	No	112 (46.5)	40 (49.4)	33 (41.2)	39 (48.8)		
Coffee consumption status—*N* (%)	Decaffeinated only	6 (2.5)	1 (1.2)	2 (2.5)	3 (3.8)	0.929	0.147
	Current consumer	179 (74.3)	62 (76.5)	60 (75.0)	57 (71.2)		
	Former consumer	22 (9.1)	6 (7.4)	7 (8.8)	9 (11.2)		
	Non- consumer	34 (14.1)	12 (14.8)	11 (13.8)	11 (13.8)		
Tea consumption status—*N* (%)	Current consumer	101 (41.9)	32 (39.5)	36 (45.0)	33 (41.2)	0.970	0.075
	Former consumer	6 (2.5)	2 (2.5)	2 (2.5)	2 (2.5)		
	Non-consumer	134 (55.6)	47 (58.0)	42 (52.5)	45 (56.2)		
Alcohol-drinking status—*N* (%)	Current drinker	157 (65.1)	52 (64.2)	59 (73.8)	46 (57.5)	0.054	0.321
	Former drinker	18 (7.5)	5 (6.2)	2 (2.5)	11 (13.8)		
	Non-drinker	66 (27.4)	24 (29.6)	19 (23.8)	23 (28.7)		
Cigarette smoking status—*N* (%)	Current smoker	28 (11.6)	10 (12.3)	10 (12.5)	8 (10.0)	0.403	0.188
	Former smoker	93 (38.6)	28 (34.6)	27 (33.8)	38 (47.5)		
	Non-smoker	120 (49.8)	43 (53.1)	43 (53.8)	34 (42.5)		
Current consumers of both coffee and tea—*N* (%)	Yes	72 (29.9)	25 (30.9)	25 (31.2)	22 (27.5)	0.850	0.055
	No	169 (70.1)	56 (69.1)	55 (68.8)	58 (72.5)		

*Missing values were excluded from the analysis and percentages were computed out of the total number of observations. p-values from ANOVA models or Chi-Square statistics for continuous and categorical variables, respectively. SMD, standardized mean difference (i.e., the average of all possible standardized mean differences)*.

* *ANOVA model and SMD were computed on log values. Tertiles of ΔFS distribution are: ≤ 0.333 (I); 0.334 – 0.875 (II); >0.875 (III)*.

### Coffee Consumption

**Figure 1:**
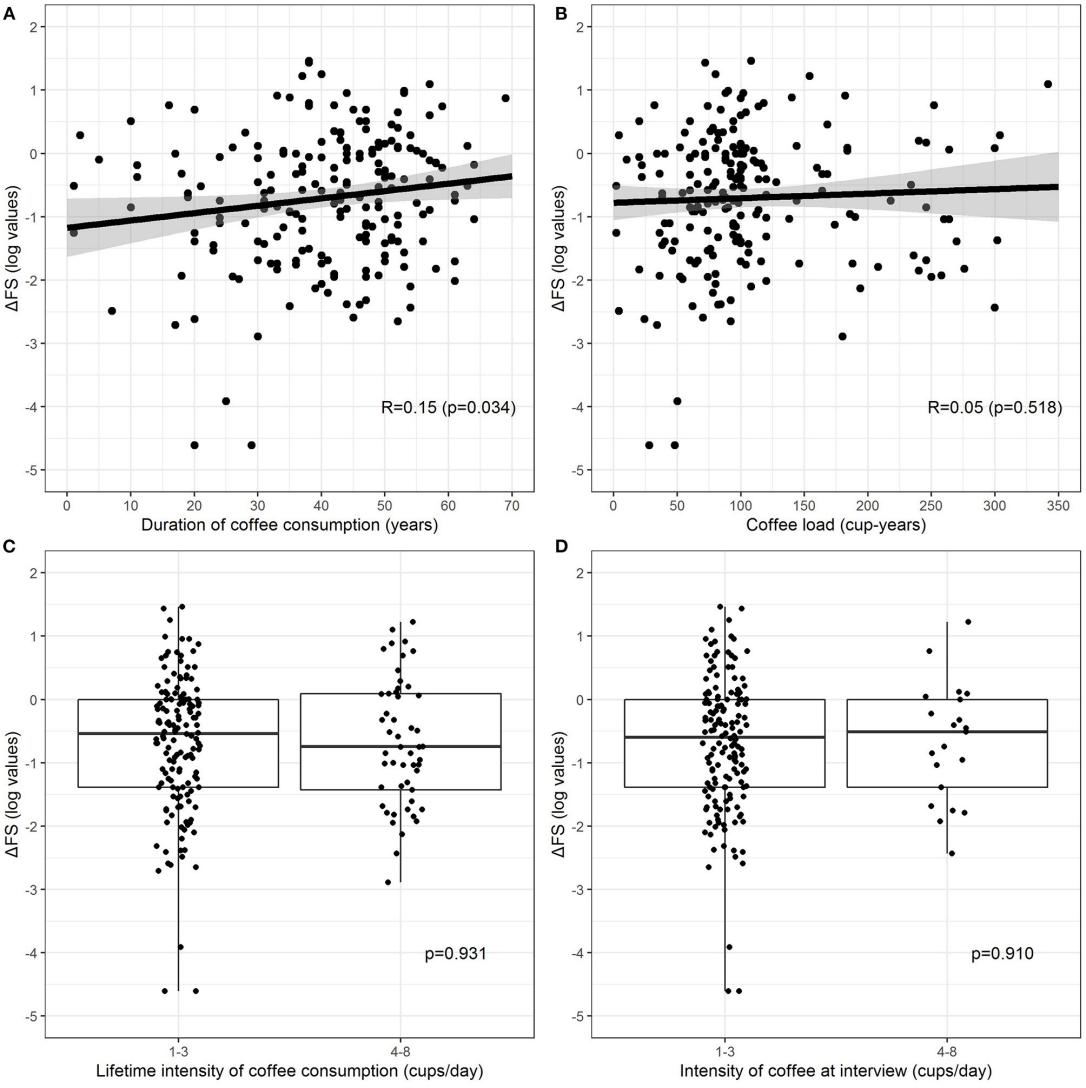
Relationship between log-transformed ΔFS and duration of coffee consumption **(A)**, coffee lifetime load **(B)**, lifetime intensity **(C)** (current and former consumers combine), and intensity at the interview **(D)** (only current consumers). Patients who consumed decaffeinated coffee were excluded. The relationship between continuous exposures and log-transformed ΔFS **(A,B)** is graphically represented by a scatterplot with a fitted regression line, along with estimated Pearson correlation coefficient (*R*) and *p*-value, whereas the association between categorical exposures and log-transformed ΔFS **(C,D)** is graphically represented by a boxplot, along with *p*-values from the two-sample *t*-test.

Current coffee consumers were 179 (74.3%), 34 (14.1%) were non-consumers, and 22 (9.1%) former consumers, whereas six patients (2.5%) consumed decaffeinated coffee only. No patients started consuming coffee after ALS diagnosis. Table 2 shows unadjusted comparisons of clinical variables among non-consumers, former consumers, and consumers of coffee according to the number of the mean daily cups during lifetime categories. Patients who consumed decaffeinated coffee only were excluded from the analysis because of their small number. The median ΔFS score was similar among all categories. All clinical factors (age, gender, age at onset, BMI, FVC) were equally distributed across the categories. The intensity at the interview was lower than the lifetime intensity, showing a reduction in coffee consumption as the disease progresses. Pairwise associations between cup-years, cups/day, duration of coffee consumption, and log-transformed ΔFS were assessed, and results are reported in Figure 1. The log-ΔFS was weakly correlated with the duration of coffee consumption (*r* = 0.15, *p* = 0.034), but not with the number of cup-years, or the intensity of coffee consumption.

**Table 2:** Clinical variables according to coffee consumption status (i.e., none vs. former vs. current consumers) and lifetime intensity of coffee consumption (i.e., mean daily cups per day groups).

**Variable**	**Category**	**Non- consumers (0 cups/day) (*N* = 34)**	**Lifetime intensity in former coffee consumers**		**Lifetime intensity in current coffee consumers**		**Comparisons (** ***p*** **-values)**			
			1–3 cups/day^*^ (*N* = 12)	4–8 cups/day^*^ (*N* = 10)	1–3 cups/day^*^ (*N* = 138)	4–8 cups/day^*^ (*N* = 41)	**1–3 vs. 4–8 cups/day among former- consumers**	**1–3 vs. 4–8 cups/day among current consumers**	**Former consumers vs. non- consumers**	**Current consumers vs. non- consumers**
Country—*N* (%)	Italy	23 (67.6)	9 (75.0)	9 (90.0)	120 (87.0)	39 (95.1)	0.594	0.171	0.309	0.003
	Moldova/ Romania	11 (32.4)	3 (25.0)	1 (10.0)	18 (13.0)	2 (4.9)				
Gender—*N* (%)	Males	18 (52.9)	7 (58.3)	6 (60.0)	86 (62.3)	26 (63.4)	1.000	1.000	0.785	0.339
	Females	16 (47.1)	5 (41.7)	4 (40.0)	52 (37.7)	15 (36.6)				
BMI at interview—*N*(%)	<18.5	5 (14.7)	1 (8.3)	2 (20.0)	7 (5.1)	0 (0.0)	0.427	0.116	1.000	0.057
	18.5–24.9	15 (44.1)	7 (58.3)	3 (30.0)	76 (55.1)	18 (43.9)				
	≥25	14 (41.2)	4 (33.3)	5 (50.0)	55 (39.9)	23 (56.1)				
Age at interview (years)	Mean ± SD	64.3 ± 11.3	64.6 ± 12.4	58.9 ± 10.1	62.5 ± 11.3	60.9 ± 9.3	0.227	0.415	0.403	0.232
Age at onset (years)	Mean ± SD	60.5 ± 12.6	60.8 ± 15.8	56.1 ± 9.3	60.6 ± 12.0	57.8 ± 9.1	0.348	0.190	0.521	0.562
Diagnostic delay (years)^#^	Median (range)	0.8 [0.1–15.8]	1.0 [0.3–4.3]	0.6 [0.3–1.8]	0.9 [0.1–5.0]	0.9 [0.1–9.3]	0.077	0.614	0.920	0.977
Education (years)	Mean ± SD	9.4 ± 4.5	12.2 ± 4.7	9.4 ± 3.9	10.3 ± 4.1	11.1 ± 5.2	0.142	0.290	0.236	0.105
Age at start coffee consuming (years)	Mean ± SD	—	22.8 ± 12.2	20.0 ± 8.5	22.1 ± 9.1	18.1 ± 5.5	0.459	0.010	—	—
Duration of coffee consumption (years)	Mean ± SD	—	39.8 ± 18.3	37.0 ± 13.9	40.4 ± 13.8	42.8 ± 10.5	0.634	0.318	—	—
Coffee load (cup-years)^#^^,^ ^§^	Median (range)	—	84.9 [4.0–119.9]	159.9 [60.0–303.8]	81.9 [2.0–119.9]	187.9 [77.9–341.8]	<0.001	<0.001	—	—
Coffee intensity at interview—*N* (%)	1–3 cups/day	—	—	—	132 (96.4)	24 (60.0)	–	<0.001	—	—
	4–8 cups/day				5 (3.6)	16 (40.0)				
ΔFS^#^	Median (range)	0.6 [0.1–5.3]	0.6 [0.1–1.5]	1.1 [0.1–2.4]	0.6 [0.0–4.3]	0.4 [0.1–3.4]	0.267	0.646	0.746	0.716

*Patients who consumed decaffeinated coffee were excluded from the analysis*.

*Missing values were excluded from the analysis and percentages were computed out of the total number of observations. SD, standard deviation; p-values from ANOVA models or Fisher exact test for continuous and categorical variables, respectively*.

# *log-transformed variable was used in the ANOVA model*.

§ *The cup-years is the unit for measuring the amount a person has consumed over a long period of time and it was computed by dividing the cumulative lifetime exposure load by 365.25 units*.

* *Lifetime intensity of coffee consumption was computed as the weighted mean of the number of cups consumed per day at different age periods, with weights equal to the duration of coffee consumption within each age period. Coffee intensity at the interview is the number of cups/day drunk at the time of the interview*.

### Tea Consumption

**Figure 2:**
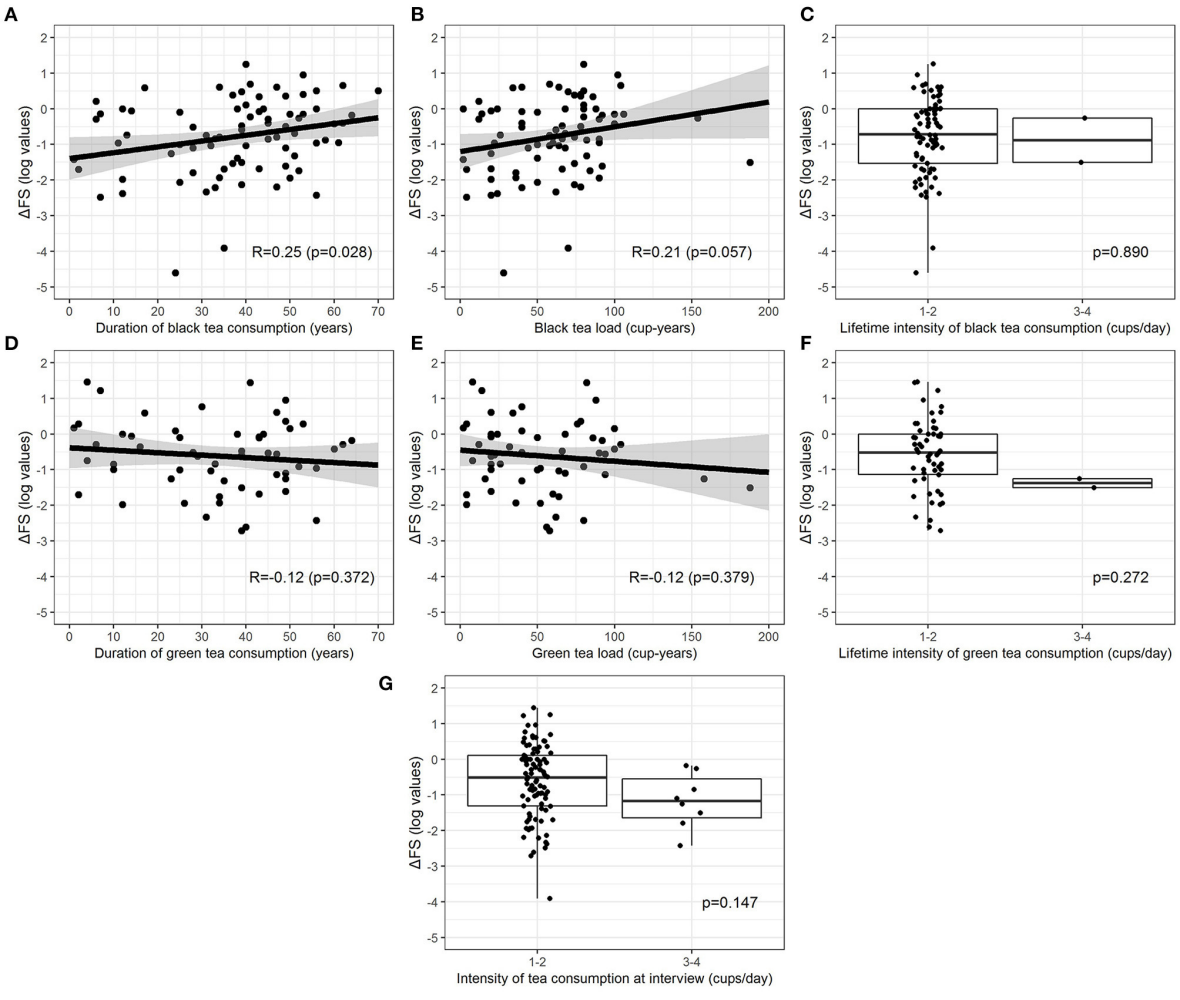
Relationship between log-transformed ΔFS and duration of consumption **(A,D)**, lifetime load **(B,E)**, and lifetime intensity **(C,F)** for green and black tea (current and former consumers). Relationship between log-transformed ΔFS and intensity at the interview for all types of tea combined **(G)** (only current consumers). The relationship between continuous exposures and log-transformed ΔFS is graphically represented by a scatterplot with a fitted regression line, along with estimated Pearson correlation coefficient (*R*) and *p*-value, whereas the association between categorical exposures and log-transformed ΔFS is graphically represented by a boxplot, along with *p*-values from the two-sample *t*-test.

Current tea consumers were 101 (41.9%), 6 (2.5%) patients were former consumers, and 134 (55.6%) were non-consumers. Among 107 current and former consumers, 27 (25.2%) consumed only green tea, 51 (47.7%) only black tea, and 29 (27.1%) consumed both. No patients started consuming tea after ALS diagnosis. Table 3 shows unadjusted comparisons of clinical variables among tea consumers, non-consumers, and former consumers according to the number of the mean daily cups during lifetime categories. The median ΔFS score was similar among all categories. We found no significant differences in the rate of disease progression between tea consumers and non-consumers. All clinical factors were equally distributed across the categories. Pairwise associations between cup-years, cups/day, duration of tea consumption, and log-transformed ΔFS were assessed, and results are reported in Figure 2. Log-ΔFS was weakly correlated only with the duration of consumption of black tea (*r* = 0.25, *p* = 0.028).

**Table 3:** Clinical variables according to tea consumption status (i.e., none vs. former vs. current consumers).

**Variable**	**Category**	**Non- consumers (*N* = 134)**	**Former tea consumers (*N* = 6)**	**Current tea consumers (*N* = 101)**	**Comparisons (** ***p*** **-values)**			
					**Overall**	**Former consumers vs. non-consumers**	**Current consumers vs. non-consumers**	**Current consumers vs. former-consumers**
Country—*N* (%)	Italy	128 (95.5)	6 (100.0)	72 (71.3)	<0.001	1.000	<0.001	0.187
	Moldova/ Romania	6 (4.5)	0 (0.0)	29 (28.7)				
Gender—*N* (%)	Males	87 (64.9)	2 (33.3)	56 (55.4)	0.154	0.190	0.177	0.409
	Females	47 (35.1)	4 (66.7)	45 (44.6)				
BMI at interview—*N* (%)	<18.5	6 (4.5)	1 (16.7)	8 (7.9)	0.123	0.423	0.097	0.333
	18.5–24.9	62 (46.3)	2 (33.3)	57 (56.4)				
	≥25	66 (49.3)	3 (50.0)	36 (35.6)				
Age at interview (years)	Mean ± SD	64.2 ± 10.5	55.7 ± 13.0	60.4 ± 11.1	0.009	0.058	0.007	0.300
Age at onset (years)	Mean ± SD	61.5 ± 11.1	51.9 ± 14.1	58.3 ± 12.2	0.028	0.048	0.038	0.188
Diagnostic delay (years)^#^	Median (range)	0.9 [0.1–15.8]	0.8 [0.2–3.0]	0.8 [0.1–9.3]	0.588	0.657	0.326	0.895
Education (years)	Mean ± SD	10.2 ± 4.7	11.7 ± 4.7	10.7 ± 3.9	0.521	0.419	0.362	0.605
Age at start tea (green or black) consuming (years)	Mean ± SD	—	24.8 ± 13.6	23.8 ± 18.5	0.893	—	—	0.893
Duration of black tea consumption (years)^#^	Median (range)	—	12.0 [0.0–31.0]	35.0 [0.0–70.0]	0.143	—	—	0.143
Duration of green tea consumption (years)^#^	Median (range)	—	2.0 [0.0–45.0]	2.0 [0.0–64.0]	0.849	—	—	0.849
Lifetime intensity of black tea consumption—*N* (%)	0 cups/day^*^	—	3 (50.0)	24 (23.8)	0.265	—	—	0.265
	1–2 cups/day^*^		3 (50.0)	75 (74.3)				
	3–4 cups/day^*^		0 (0.0)	2 (2.0)				
Lifetime intensity of green tea consumption—*N* (%)	0 cups/day^*^	—	3 (50.0)	48 (47.5)	1.000	—	—	1.000
	1–2 cups/day^*^		3 (50.0)	51 (50.5)				
	3–4 cups/day^*^		0 (0.0)	2 (2.0)				
Black tea load (cup-years)^#^^,^ ^§^	Median (range)	—	14.0 [0.0–58.0]	50.0 [0.0–187.9]	0.152	—	—	0.152
Green tea load (cup-years)^#^^,^ ^§^	Median (range)	—	4.0 [0.0–89.9]	4.0 [0.0–187.9]	0.926	—	—	0.926
Tea intensity (green or black) at interview (cups/day)	1–2 cups/day	—	—	93 (92.1)	—	—	—	—
	3–4 cups/day			8 (7.9)				
ΔFS^#^	Median (range)	0.6 [0.0–5.3]	0.5 [0.0–4.3]	0.6 [0.0–4.2]	0.639	0.356	0.931	0.345

*Missing values were excluded from the analysis and percentages were computed out of the total number of observations. SD, standard deviation; p-values from ANOVA models or Fisher exact test for continuous and categorical variables, respectively*.

# *log-transformed variable was used in the ANOVA model*.

§ *The cup-years is the unit for measuring the amount a person has consumed over a long period of time and it was computed by dividing the cumulative lifetime exposure load by 365.25 units*.

* *Lifetime intensity of tea consumption was computed as the weighted mean of the number of cups consumed per day at different age periods, with weights equal to the duration of tea consumption within each age period. Tea intensity at the interview is the number of cups/day drunk at the time of the interview*.

### Predictors of ΔFS

The VIMP provided by the conditional RF algorithm that we used to detect the variables most associated with (log-transformed) ΔFS suggested that age at onset, education, and site of onset were covariates, which explained the largest amount of the log-ΔFS variance (Table 4). Specifically, the age at onset was the strongest predictor, achieving the highest VIMP of 0.21 at the top of VIMP list, whereas all the lifestyle variables had VIMPs close to 0. The association between coffee and tea consumption (measured in cups/day both as a lifetime weighted average and “at interview”) on the log ΔFS was eventually assessed in univariable and multivariable analyses, adjusting ANOVA models for the six possible confounders (age at onset, gender, country, education, alcohol drinking, and smoking status), alone or in combination. Results are reported in Table 5; log-ΔFS least-square means did not significantly vary across coffee and tea consumption groups.

**Table 4:** Variable importance (VIMP) and relative variable importance (RVIMP) values from conditional Random Forest algorithm (100,000 trees) of each candidate clinical, demographical, pathological, treatment, and coffee/tea consumption variables in explaining the variability of the ΔFS (log values).

**Rank**	**Variable**	**VIMP**	**RVIMP**
1	Age at onset	0.2075	100.00%
2	Education	0.0298	14.34%
3	Site of onset	0.0118	5.67%
4	Country	0.0058	2.79%
5	Duration of coffee consumption	0.0049	2.36%
6	Current alchool drinker	0.0048	2.32%
7	Lifetime intensity of green tea consumption (cups/day)	0.0016	0.78%
8	Gender	0.0009	0.45%
9	BMI	0.0008	0.37%
10	Lifetime intensity of coffee consumption (cups/day)	0.0006	0.28%
11	Other types of tea load (cup-years)	0.0003	0.16%
12	Duration of other tea consumption	0.0001	0.06%
13	Lifetime intensity of other tea consumption (cups/day)	0.0000	0.00%
14	Tea consumption status	0.0000	0.00%
15	Green tea load (cup-years)	0.0000	0.00%
16	Tea intensity at interview	0.0000	0.00%
17	Duration of green tea consumption	0.0000	0.00%
18	Coffee load (cup-years)	0.0000	0.00%
19	Coffee consumption status	0.0000	0.00%
20	Riluzole	0.0000	0.00%
21	Current smokers	0.0000	0.00%
22	Coffee intensity at interview	0.0000	0.00%

*Variables are ranked from the most to the less important (rank)*.

*VIMP is the sum of the decrease in prediction (of log-ΔFS) error values when a tree split by that variable whereas RVIMP is the VIMP divided by the highest VIMP value so that values are bounded between 0 and 1 (or between 0 and 100%)*.

**Table 5:** Association between the intensity of coffee and tea consumption on ΔFS, unadjusted and adjusted least-square means from ANOVA models using ΔFS log-transformed values.

		ΔFS Least square means (95%CI)^*^			
**Exposure**	**Confounders**	**Group 1**	**Group 2**	**Group 3**	***p*-value**
Lifetime intensity of coffee consumption (groups) 0 cups/day (*N* = 34) 1–3 cups/day (*N* = 150) 4–8 cups/day (*N* = 51)	None (unadjusted)	0.52 (0.36–0.74)	0.49 (0.42–0.59)	0.50 (0.37–0.68)	0.978
	Age at onset	0.50 (0.36–0.71)	0.48 (0.41–0.57)	0.55 (0.42–0.72)	0.975
	Gender	0.52 (0.36–0.75)	0.50 (0.42–0.60)	0.51 (0.38–0.69)	0.978
	Country	0.54 (0.37–0.78)	0.53 (0.43–0.67)	0.55 (0.39–0.78)	0.978
	Education	0.49 (0.34–0.71)	0.50 (0.42–0.59)	0.51 (0.38–0.69)	0.978
	Alcoholic-drinking status	0.56 (0.38–0.82)	0.55 (0.44–0.69)	0.56 (0.40–0.78)	0.978
	Cigarette smoking status	0.52 (0.35–0.77)	0.48 (0.40–0.59)	0.47 (0.35–0.65)	0.978
Lifetime intensity of green tea consumption (groups) 0 cups/day (*N* = 185) 1–2 cups/day (*N* = 54) 3–4 cups/day (*N* = 2)	None (unadjusted)	0.49 (0.42–0.58)	0.56 (0.42–0.74)	0.25 (0.06–1.13)	0.505
	Age at onset	0.48 (0.42–0.56)	0.59 (0.45–0.78)	0.30 (0.07–1.20)	0.453
	Gender	0.50 (0.43–0.59)	0.56 (0.42–0.74)	0.24 (0.05–1.07)	0.506
	Country	0.54 (0.43–0.69)	0.58 (0.43–0.77)	0.22 (0.05–1.02)	0.505
	Education	0.49 (0.42–0.58)	0.55 (0.42–0.74)	0.25 (0.06–1.11)	0.496
	Alcoholic-drinking status	0.55 (0.44–0.68)	0.61 (0.45–0.85)	0.29 (0.06–1.30)	0.505
	Cigarette smoking status	0.48 (0.40–0.58)	0.56 (0.41–0.77)	0.28 (0.06–1.25)	0.503
Lifetime intensity of other types of tea consumption (groups) 0 cups/day (*N* = 161) 1–2 cups/day (*N* = 78) 3–4 cups/day (*N* = 2)	None (unadjusted)	0.53 (0.45–0.62)	0.46 (0.36–0.59)	0.41 (0.09–1.86)	0.642
	Age at onset	0.51 (0.44–0.60)	0.49 (0.39–0.61)	0.44 (0.11–1.79)	0.600
	Gender	0.54 (0.45–0.64)	0.46 (0.36–0.59)	0.41 (0.09–1.86)	0.642
	Country	0.58 (0.46–0.73)	0.50 (0.38–0.65)	0.36 (0.08–1.66)	0.641
	Education	0.52 (0.44–0.62)	0.47 (0.37–0.60)	0.43 (0.10–1.91)	0.635
	Alcoholic-drinking status	0.59 (0.47–0.74)	0.51 (0.38–0.67)	0.48 (0.10–2.16)	0.642
	Cigarette smoking status	0.51 (0.42–0.62)	0.46 (0.35–0.60)	0.45 (0.10–2.03)	0.641
Coffee intensity at interview (groups) 1–3 cups/day (*N* = 156) 4–8 cups/day (*N* = 21)	None (unadjusted)	0.49 (0.42–0.58)	0.51 (0.32–0.80)	—	0.910
	Age at onset	0.49 (0.42–0.57)	0.58 (0.38–0.87)	—	0.902
	Gender	0.50 (0.43–0.60)	0.52 (0.33–0.82)	—	0.910
	Country	0.55 (0.43–0.71)	0.58 (0.35–0.96)		0.910
	Education	0.49 (0.42–0.58)	0.51 (0.33–0.80)	—	0.909
	Alcoholic-drinking status	0.60 (0.46–0.78)	0.57 (0.36–0.92)	—	0.909
	Cigarette smoking status	0.48 (0.40–0.58)	0.49 (0.31–0.77)	—	0.91
Tea (green or other types) intensity at interview (groups) 1–2 cups/day (*N* = 93) 3–4 cups/day (*N* = 8)	None (unadjusted)	0.54 (0.43–0.66)	0.31 (0.15–0.63)	—	0.147
	Age at onset	0.53 (0.44–0.64)	0.37 (0.19–0.71)	—	0.114
	Gender	0.54 (0.44–0.66)	0.31 (0.15–0.63)	—	0.149
	Country	0.58 (0.45–0.74)	0.27 (0.13–0.57)		0.146
	Education	0.54 (0.43–0.66)	0.31 (0.15–0.63)	—	0.149
	Alcoholic-drinking status	0.57 (0.43–0.77)	0.33 (0.16–0.69)	—	0.149
	Cigarette smoking status	0.53 (0.41–0.69)	0.34 (0.16–0.72)	—	0.148

* *Least square means along with 95% confidence interval (CI) were exponentiated for ease of interpretation*.

## Discussion

We found no correlation between coffee or tea consumption and disease progression. Log-ΔFS was only weakly correlated with the duration of coffee and black tea consumption, but not with the number of cup-years or cups/day. Coffee and tea consumptions have been studied in ALS for their possible role in the risk of developing the disease, but their possible role as predictors of the disease course once it has begun has not been evaluated so far. A pooled analysis based on over 1,000,000 individuals from five cohorts (8) did not show an association of caffeine and tea intakes with the risk of dying from ALS. A pooled analysis of eight international prospective cohort studies, including 351,565 individuals (9), did not observe statistically significant associations between coffee, tea, or caffeine intake and ALS mortality risk. Only one study was in contrast with these observations (21), showing that coffee intake was less frequent and prolonged among ALS patients than in different groups of sick or healthy controls. However, the odds for exposure among ALS patients decreased after excluding cases and controls who stopped consuming coffee after disease onset, and an exposure gradient was not detected. This study also found a small, although significant protective effect of smoking, which is also in contrast with most studies (22), suggesting the possibility of bias. A case–control study conducted in almost the same population some years later (23) did not confirm these data but found a small risk reduction for tea.

To analyze the possible role of beverages on disease progression, we divided log-ΔFS into tertiles. Tertiles of ΔFS distribution are associated with survival (13, 24), indicating that this measure predicts different disease progressions. Slow progressors had a younger age at disease onset, more frequent spinal onset, better FVC%, and longer diagnostic delay, all positive predictive factors for ALS progression. Coffee and tea consumption status was equally distributed across progression categories. ΔFS score and age at disease onset were substantially similar for coffee, green and black tea, across consumption categories. All these findings are against a role for coffee or tea in influencing disease progression, in analogy with cohort studies indicating that coffee and tea intakes are not a risk factor for disease susceptibility. A few experimental studies do not help to understand the role of coffee in ALS. Chronic caffeine intake significantly reduced survival in superoxide dismutase 1 G93A mice, an animal model of ALS (25), but in another study, coffee improved motor performance of male G93A mice (26).

To analyze a possible interaction of coffee and tea consumptions with other lifestyle factors and clinical variables, we firstly ranked variables, using a variable importance measure, and eventually performed a multivariable model. None of the lifestyle variables analyzed was ranked high. Clinical/demographic variables, such as age at onset, site of onset, and education, explained the largest amount of log-ΔFS variance. Adjusting for potential clinical and demographic confounders, the multivariable analysis did not show any association between coffee and tea consumption and log ΔFS.

The limitations of our study are related to a possible recall bias, which seems improbable given that patients were unaware of the study hypothesis, and interviewers were blinded to clinical history and neurological status. We could not evaluate the influence of unmeasured variables, such as physical activity, trauma, and diet, but it is unlikely that these are confounders of coffee or tea consumption.

Although the findings should be interpreted with caution, this study has several strengths. Selection bias was minimized because patients were consecutively enrolled at five different Centers and included a large spectrum of disease severity. Previous cohort and case–control studies only assessed the baseline intake of coffee and tea, but not the personal history of consumption for every single patient. On the contrary, we studied both the exposures at the time of the interview, and their lifetime cumulative effect, using a cup-year measure in analogy to pack-year research on smoking.

Our study does not support the hypothesis that coffee or tea intake is associated with a different ALS progression, contrarily to other neurodegenerative diseases. Although our findings seem rather strong, we cannot exclude a possible effect of coffee or tea on a subgroup of patients, for example, with positive family history. However, this could only be studied with a much larger sample of patients.

## Data Availability Statement

The raw data supporting the conclusions of this article will be made available by the authors, without undue reservation.

## Ethics Statement

The studies involving human participants were reviewed and approved by Boards of the coordinating Center (N96/CE/2016). The patients/participants provided their written informed consent to participate in this study.

## Author Contributions

AC: study design, recruitment, examination of patients, data collecting, management, analysis, final approval of the version to be published, drafting the paper, and agreement to be accountable for all aspects of the work in ensuring that questions related to the accuracy or integrity of any part of the work are appropriately investigated and resolved. AI: study design, management, analysis, final approval of the version to be published, drafting the paper, and writing the paper. AF: study design, data analysis, data interpretation, and drafting the paper. SR: data set, data analysis, and data interpretation. LM: conception of the study, recruitment, examination of patients, data collecting, and writing. JM: recruitment, examination of patients, data collecting, and final approval of the version to be published. VL and DM: contribution to the conception of the study, final approval of the version of the manuscript to be published, and revising critically the research and the manuscript. ML: study design, recruitment, data management, analysis, drafting the paper, and final approval of the version to be published. All authors contributed to the article and approved the submitted version.

## Conflict of Interest

The authors declare that the research was conducted in the absence of any commercial or financial relationships that could be construed as a potential conflict of interest.

## Publisher's Note

All claims expressed in this article are solely those of the authors and do not necessarily represent those of their affiliated organizations, or those of the publisher, the editors and the reviewers. Any product that may be evaluated in this article, or claim that may be made by its manufacturer, is not guaranteed or endorsed by the publisher.
